# Subjective Health Literacy and Personality in Older Adults: Conscientiousness, Neuroticism, and Openness as Key Predictors—A Cross-Sectional Study

**DOI:** 10.3390/ijerph22030392

**Published:** 2025-03-07

**Authors:** Lena Haarmann, Elke Kalbe, Görkem Anapa, Dilara Kurt, Ümran Sema Seven

**Affiliations:** Medical Psychology|Neuropsychology and Gender Studies & Center for Neuropsychological Diagnostics and Intervention (CeNDI), Faculty of Medicine and University Hospital Cologne, University of Cologne, 50937 Cologne, Germany; lena.haarmann@uk-koeln.de (L.H.); elke.kalbe@uk-koeln.de (E.K.); goerkem.anapa@gmail.com (G.A.); dilara.kurt@posteo.de (D.K.)

**Keywords:** health literacy, HLS-EU-Q47, personality, Big Five personality traits

## Abstract

Low health literacy (HL) is associated with numerous negative health behaviors and outcomes, making it crucial to understand its underlying determinants. While associations between sociodemographic variables and subjective HL have already been demonstrated, data on the association between HL and personality remain limited. This study aims to extend the current knowledge by exploring how personality traits influence HL, beyond the effects of sociodemographic variables on HL. A cross-sectional study was performed with a sample of 238 healthy participants aged 50 to 92 years. Personality was measured using the NEO Five-Factor Inventory and subjective health literacy using the HLS-EU-Q47 questionnaire. Descriptive and correlational analyses as well as a multiple linear regression analysis with the Big Five personality traits, sex, age, and education as predictors of subjective health literacy were performed. The General-HL index was 37.22 (SD 7.98), which corresponds to sufficient or non-limited health literacy. The Big Five personality traits accounted for 32.2% of the variance in health literacy. Of the traits, Conscientiousness emerged as the strongest predictor (β = 0.31, medium effect), followed by Neuroticism (β = −0.21, small effect) and Openness to experience (β = 0.15, small effect). Sex was also a significant predictor of health literacy (β = 0.14, small effect). These results suggest that personality plays a significant role in health literacy, with higher Conscientiousness, lower Neuroticism, and higher Openness to experience, as well as female sex, predicting better health literacy. These findings underscore the importance of considering personality traits in interventions aimed at improving health literacy, with potential implications for both theoretical understanding and practical application in healthcare settings.

## 1. Introduction

In recent decades, the concept of health literacy (HL) has developed steadily; although, originally, research on HL focused predominantly on functional abilities (basic linguistic and mathematical skills in medical contexts), the concept has continuously expanded, now encompassing various elements of empowerment, such as communicating and navigating successfully within complex healthcare systems [1]. To standardize the numerous approaches of definitions an international team of experts (HLS-EU consortium) developed a comprehensive model that defines HL as “people’s knowledge, motivation and competences to access, understand, appraise, and apply health information in order to make judgments and take decisions in everyday life concerning healthcare, disease prevention and health promotion to maintain or improve quality of life during the life course” [2]. Based on this definition, a new instrument, namely, the HLS-EU Scale, was developed to overcome the weakness of previous instruments, which had focused mainly on functional abilities [3,4]. In accordance with the established definition, the scale measures the subjectively perceived difficulty of health-related tasks concerning healthcare, disease prevention, and health promotion via 47 items [4]. Since its introduction, the HLS-EU Scale has been used widely but has also been criticized for assessing HL only subjectively [5]. Nevertheless, HL, assessed subjectively via the HLS-EU Scale, has been associated with health-related outcomes, emphasizing the importance of subjective HL both for the individual and for healthcare systems. For instance, the age-related increase in health service utilization is smallest for individuals with high health literacy scores and high levels of health-related knowledge [6]. A representative study of 2000 participants found that of those who demonstrated excellent subjective HL, 95.1% rated their health as “good” or “very good”; while among those respondents who demonstrated inadequate health literacy, only 43.8% rated their own health positively [7]. Health literacy is essential for both individual health and the efficiency of healthcare systems. It empowers individuals to make informed decisions, reducing preventable health issues and healthcare costs. Improving health literacy also helps address health disparities as those with higher health literacy are more likely to adopt healthier behaviors and seek timely care. Ultimately, promoting health literacy supports healthier populations and more sustainable healthcare systems [7] (Schaeffer et al., 2016). With regard to health behavior (nutritional behavior, physical activity, body mass index, and medication intake), positive correlations between subjective HL and health-promoting behavior have been found [7]. Another study found that well-being and self-assessed health status were more strongly associated with subjective HL than with health-related knowledge [3], suggesting that in the sense of a self-fulfilling prophecy, the individual’s perception of health-related competence might even outweigh his or her actual knowledge about health-related issues. Furthermore, the study revealed that subjective HL and health-related knowledge were only weakly correlated [3]. If knowledge is not the only major factor in explaining subjective HL, what other factors are decisive in determining high subjective HL? Gerich and Moosbrugger (2018) explored individual experiences and factors and their associations with HLS-EU Scale-scores [3]. They identified four types of respondents according to their scores on the following two dimensions: health-related knowledge and subjective HL (high and low scores of both dimensions were split at the median). The two types that were high in subjective HL were characterized by either high levels of personal and social resources such as high self-efficacy, active coping, self-medication, and less fatalistic attitudes (type of respondents scoring high on health-related knowledge) or by a high level of trust in doctors and the health systems and an external locus of control (type of respondents scoring low on health-related knowledge) [3]. Therefore, the authors concluded that “subjective HL is a measure of how patients perceive the manageability of different health-related tasks” [3]. High manageability can result from two independent factors: personal resources and skills (e.g., self-efficacy and active coping style) or trust and adherence to health professionals’ instructions. However, the authors also suggest an alternative explanation of reverse causality: limited experience with health issues might affect personal resources, trust, and adherence, thereby, in turn, affecting subjective HL [3]. To expand the rationale behind the knowledge of respondents’ answers on the HLS-EU Scale, we aim to extend the approach of Gerich and Moosbrugger (2018) by personal factors that are less likely to be ruled out by this alternative explanation as they are less alterable by personal experience and are thus less prone to reverse causality [3]. By incorporating personality traits, which are relatively stable over time, our approach goes beyond the sociodemographic factors previously considered. This adds a new layer to understanding subjective health literacy, highlighting the role of inherent personal characteristics in shaping individuals’ perceptions of health-related tasks.

We focus on personality (Big Five) [8] and sociodemographic variables (sex, age, and education) to better understand response patterns measured by the HLS-EU Scale. Since the impact of sociodemographic factors on subjective HL is already well established—showing associations with female sex, younger age, and higher education [9,10,11]—our main focus is on the additional effects of personality.

Regarding personality, the “Big Five model” [8] is the most common model for classification. According to the model, personality can be described by the means of five dimensions: Neuroticism, which is the tendency to experience negative emotional states, e.g., anxiety, anger, and guilt [12]; Extraversion, i.e., the tendency “to experience and exhibit positive affect, assertive behavior, decisive thinking, and desires for social attention’ [13]; Agreeableness, which can be described as ‘the motivation to maintain positive relations with others’ [14]; Openness to experience, which describes being curious and having a wide range of interests and a need for novelty and variety [15]; and finally, Conscientiousness, i.e., ‘the propensity to be self-controlled, responsible to others, hardworking, orderly, and rule-abiding” [16].

How are these five personality dimensions linked to manageability (in terms of (a) personal resources, e.g., self-efficacy or active coping, and (b) trust and adherence) and contribute to shaping the respondent’s answers on health-related tasks? (a) In various contexts, personality traits have been linked to personal resources. Neuroticism negatively correlates with self-efficacy, while Extraversion, Conscientiousness, and Agreeableness show positive associations [17]. Similar patterns exist for self-esteem, with Neuroticism being negatively correlated and Extraversion and Conscientiousness being positively correlated [18]. Regarding coping, Conscientiousness and Openness are positively associated with self-perceived coping abilities and active coping (i.e., tackling a task directly), whereas Neuroticism correlates negatively and is linked to defensive coping (i.e., avoiding thinking about a task) [19]. (b) In terms of trust, adherence, and personality dimensions, studies have shown that the Big Five traits influence health-related behaviors. A prospective study on ACL rehabilitation (anterior cruciate ligament reconstruction surgery) found that Agreeableness predicted attendance, while Openness and Conscientiousness predicted adherence (rated by rehabilitation professionals) [20]. Another study linked Neuroticism (negative) and Conscientiousness (positive) to diabetes management adherence, with small positive correlations for Extraversion and Agreeableness [21]. Conscientiousness was also associated with adherence to renal dialysis medication [22], and its positive impact on medication adherence was confirmed in a meta-analysis [23].

Another study examined the influence of demographic characteristics and personality variables on health information literacy in a sample of young adults [24]. They found Extraversion, measured with the NEO-FFI-30 [25], and education to be predictors of health information literacy. Rüegg and Abel (2019) found, using a sample of male young adults, that psychosocial covariates like Conscientiousness, Openness, and self-efficacy were significantly related to health literacy [26]. Iwasa and Yoshida (2020) also found support for a personality and health literacy association in their Japanese sample (60–84 years): Neuroticism (β = −0.08), Extraversion (β = 0.10), Openness (β = 0.17), and Conscientiousness (β = 0.09) were, among education, subjective economic status, social isolation, and functional dependence, independently and significantly associated with health literacy [27]. In a sample of Americans aged 50 and older, Kim, Zhang, and Svynarenko (2017) found that Neuroticism and Extraversion were a negative factor for health literacy [28]. A recent study in older adults from Switzerland found that after controlling for social, regional, and health characteristics, more open individuals had higher health literacy competencies, whereas individuals with higher levels of neuroticism had more difficulties with specific health-related tasks or situations [29].

Certain groups in society are at higher risk for lower subjective health literacy, including individuals with a migration background, low education, chronic diseases, and older adults [1], who require particular attention. The German Health Literacy Report identifies older adults (65+) as a particularly vulnerable group as health literacy becomes increasingly important with age due to rising health issues [7,30]. Despite this, they have the lowest health literacy: 66.3% show problematic or inadequate levels, compared to ~40% in younger groups. Only 3% of those who are 65+ demonstrate excellent health literacy [7]. This is especially critical given demographic change and an aging population [31]. To address their higher vulnerability [1] and enhance knowledge of subjective health literacy in the elderly, we focus on older adults.

The aim of this study is to extend Gerich and Moosbrugger’s (2018) approach to explore how less alterable personal resources (personality traits) shape subjective health literacy, i.e., an individual’s perception of managing health tasks, beyond sociodemographic factors (sex, age, and education) [3]. Given the growing importance of subjective health literacy, understanding its theoretical foundations and operationalization through the HLS-EU Scale is of significant public interest.

Based on prior evidence, we hypothesize that Conscientiousness will show the strongest positive association with subjective HL given its consistent links to both personal resources and trust/adherence. In contrast, we expect Neuroticism to be negatively associated with subjective HL as it has been consistently linked to lower personal resources and reduced trust and adherence. For Openness, Extraversion, and Agreeableness, existing findings are less consistent, with some studies reporting positive associations and others finding no significant effects. Given this variability, as well as the fact that these traits are theoretically less directly tied to manageability compared to Conscientiousness and Neuroticism, we anticipate at most small positive associations for these three traits with subjective HL.

## 2. Materials and Methods

### 2.1. Data Collection and Participants

This cross-sectional study was conducted at the University Hospital of Cologne. Participants were recruited between September 2017 and July 2018 in North Rhine-Westphalia, particularly in Cologne and the surrounding region. Senior citizens’ organizations were contacted via e-mail, and notices were posted in public places. As it was of interest to include healthy older adults in this study, the following inclusion and exclusion criteria were applied: Participants had to be at least 50 years old and German native speakers. The exclusion criteria were a diagnosis of dementia or a Mini-Mental State Examination (MMSE [32]) score lower than 26 and clinically relevant symptoms of depression, with a score higher than 10 on the Geriatric Depression Scale (GDS [33]). Additionally, participants were excluded if they had another severe neurological disorder affecting the central nervous system, another severe psychiatric disorder, a history of substance dependence within the past three years, or a life-threatening illness. Furthermore, individuals with severe physical or cognitive impairments that would hinder their ability to participate in this study were excluded. Participants were also required to possess sufficient hearing, reading abilities, and eyesight.

### 2.2. Measures

*Demographics of Participants*

Demographics were assessed at the beginning of this study and included age, sex, education (years of school and highest school degree), and nationality. Additionally, self-assessment questions regarding German language skills (reading, writing, and communication) as well as existing diseases, such as diabetes mellitus, high blood pressure, etc., were recorded.

*Health Literacy*

The paper-and-pencil questionnaire HLS-EU-Q47 was used to assess subjective HL [2], which was developed by the HLS-EU-Consortium [4] and consists of 47 items that are formulated as direct questions and can be answered on a four-point Likert scale (very easy, easy, difficult, and very difficult).

If a participant did not answer, he or she could tick ‘don’t know’, which was treated as a missing value [2]. Participants were asked to rate the perceived difficulty of health-relevant tasks [2]. The questionnaire assessed both individual competencies and situational demands [2]. A General-HL index and three dimension-specific indices of HL (healthcare, disease prevention, and health promotion) were calculated according to the instrument manual [2]. It is also possible to calculate information processing-specific indices [2]. The General-HL index consists of all 47 items, and the dimension-specific indices consist of 15 (disease prevention) or 16 (healthcare health promotion) items. Indices can range from 0 to 50, and answers from at least 80% of the relevant items have to be available to calculate the index [2]. In addition to the indices, there are four levels of subjective HL, which were also calculated to examine the distribution of HL in the sample: excellent (43−50 points in the General-HL index), sufficient (34−42), problematic (26−33) and inadequate (0−25) HL [2]. Previous research has shown that the HLS-Q-EU is a feasible instrument for older adults [34].

*Personality*

The NEO Five-Factor Inventory (NEO-FFI [35]) is a 60-item paper-and-pencil personality inventory that is commonly used to assess the Big Five personality traits (Neuroticism, Extraversion, Openness to experience, Agreeableness, and Conscientiousness). The NEO-FFI was used in the validated German version [35]. All items were rated using a five-point Likert scale ranging from 0 (strongly disagree) to 4 (strongly agree). There are 12 items for each scale, and scores can range from 0 to 48 for each scale.

### 2.3. Statistical Analysis

All data were analyzed using IBM SPSS Statistics (Version 24.0). The study design was cross-sectional. An a priori power analysis using G*Power [36] was conducted, indicating that a minimum of 82 participants was required for a model with 8 predictors, assuming a medium effect size (R^2^ = 0.17) based on Sørensen et al. (2015) [10], a conventional power of 0.80, and α = 0.05 [10]. The independent variables were sex, which was categorized as the ‘male’ (0) or ‘female’ (1) sex, education (years of school), age, and the Big Five personality traits. Descriptive statistics were used for all study variables and indicated as the mean and standard deviation (SD) for continuous variables and as percentages and the frequency for categorical variables. Differences between sexes for continuous variables were calculated using independent t-tests with Bonferroni-adjusted alpha levels within personality (5 comparisons, i.e., *p* = 0.01) and HL (4 comparisons, i.e., *p* = 0.0125) variables. Sex differences for categorical variables were analyzed using a chi-square test (HL level) and a Fisher’s exact test (highest school degree). The reliability of the HLS-EU-Q47 and NEO-FFI was assessed using Cronbach’s Alpha. Correlational analyses were used to determine the strength and direction of associations among the independent variables and between the outcome variable and the independent variables. A multiple hierarchical regression analysis (method: enter) was conducted to analyze the effects of the Big Five personality traits over and above sociodemographic variables (sex, age, and education) on subjective HL. The General-HL index was entered as the outcome variable. Before regression analysis, data assumptions were checked. All statistical assumptions were met. There was no multicollinearity between study variables in the regression analysis: VIF values were between 1.00 and 1.84. Linearity and homoscedasticity were also not violated (visual inspection). Normal distribution of residuals was checked with histograms and normal distribution diagrams. The residuals were normally distributed. Concerning influential data points, Cook’s Distance was not greater than 1 (max. 0.049), indicating no influential data points [37]. To identify outliers, leverage values were calculated (highest leverage value: 0.103); this value was below the threshold of 0.20 and, therefore, not problematic [38]. For all statistical analyses, a significance level of α = 5% was set, except if Bonferroni-adjusted alpha levels were used, as mentioned above. Effect sizes were interpreted as follows: The bivariate correlation coefficient was assumed to be small at 0.10, medium at 0.30, and large at 0.50 [33]. For t-tests, Cohen’s d was used and interpreted as follows: 0.20 (small effect), 0.50 (medium), and 0.80 (large effect) [37]. For regression analysis, an f2 of 0.02 was assumed to have a small effect, with a medium effect at 0.15 and a large effect above 0.35 [37].

### 2.4. Reliability and Validity

In this sample, the reliability of the general health literacy (General-HL) index was found to be exceptionally high, with a Cronbach’s Alpha coefficient of α = 0.97, indicating excellent internal consistency. This suggests that the items included in the General-HL index are highly reliable in measuring the construct of general health literacy.

For the dimension-specific indices of health literacy, reliability was also robust. The healthcare literacy index demonstrated strong internal consistency with a Cronbach’s Alpha of α = 0.92. Similarly, the disease prevention and health promotion literacy indices showed a Cronbach’s Alpha value of α = 0.93, indicating that these scales also possess excellent reliability in assessing their respective domains of health literacy.

In addition to the health literacy indices, the reliability of the Big Five personality traits was also evaluated. The Cronbach’s Alpha coefficients for these traits were generally strong, reflecting the reliability of the personality measures in this sample. Specifically, Neuroticism had a Cronbach’s Alpha of α = 0.84, indicating good internal consistency. The traits of Extraversion and Openness to experience each demonstrated a Cronbach’s Alpha of α = 0.75, while Agreeableness had a value of α = 0.77; Conscientiousness had a Cronbach’s Alpha of α = 0.81. These values suggest that the Big Five personality dimensions were measured reliably within this study, with all scales meeting acceptable reliability thresholds.

Regarding the validity of the two main variables of interest—personality traits and health literacy—it has been demonstrated through factor analyses with other personality inventories that the construct of ‘personality’ is reliably measured by the NEO-FFI. The instrument’s validity is further supported by relatively high correlations between self- and peer-assessments, ranging from r = 0.49 to r = 0.61, which reinforces the credibility of the NEO-FFI as a valid tool for measuring personality [35]. Additionally, the content and face validity of the HLS-EU-Q47 have been ensured through a rigorous and systematic process of item development and selection. This process involved an international team of experts working collaboratively in a step-by-step participatory manner [2,7], ensuring the tool’s relevance and accuracy in assessing health literacy.

## 3. Results

Complete data sets were available for the original 238 participants that could be included in the regression analysis. Data on years of school were missing for 20 participants (7.2%), and data on the General-HL index were missing for 23 participants (8.3%). Some participants had missing data on both variables, resulting in a final sample size of 238 for all following analyses.

Using “listwise deletion”, 238 of 278 participants could be included in the regression analysis. Therefore, 14.4% of the data were missing for this analysis. The descriptive statistics for all assessed participants (*n* = 278) can be found in Supporting Information Tables S1 and S2 and Figure S1. Notably, there were only minor differences in the means, SDs, and percentages compared to the 238 participants.

### 3.1. Demographics

The participants (*n* = 238) ranged from 50 to 92 years of age, with a mean age of 61.93 (SD: 10.30 years). The descriptive statistics of the sample are displayed in Table 1. The sample was sex-balanced (female sex: 52.5%). Female and male participants did not differ significantly in age, education (years of school and highest school degree), and GDS and MMSE scores. Most of the participants were highly educated: 62.9% had an “advanced technical college entrance qualification (German: Fachhochschulreife)/university entrance qualification (German: Allgemeine Hochschulreife)”. MMSE scores ranged from 26 to 30, and GDS scores ranged from 0 to 9; therefore, no participant had to be excluded.

### 3.2. Personality

Female and male participants differed significantly in Neuroticism, Openness to experience, and Agreeableness, with higher scores for women (see Table 1). In the total sample, the highest scores were found for Conscientiousness, with the lowest ones for Neuroticism (Table 1).

### 3.3. Health Literacy

**Figure 1:**
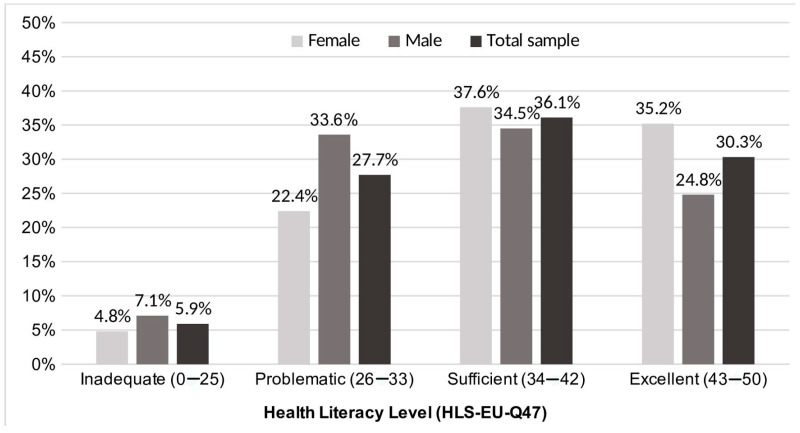
Distribution of health literacy levels (HLS-EU-Q47) in the total sample (*n* = 238) and by sex (female *n* = 125 and male *n* = 113). The classification into the levels was based on the General-HL index (score range: 0–50).

It was found that 66.4% of the participants (*n* = 158) had high subjective HL (excellent and sufficient) and that 33.6% (*n* = 80) had limited subjective HL (problematic and inadequate) (see Figure 1). There were more women than men with excellent HL, and more men in comparison to women had a problematic or inadequate subjective HL level, but the chi-square test was not statistically significant (*p* = 0.138). Female and male participants differed significantly in the health promotion literacy index, with higher values for female participants (Table 2).

### 3.4. Correlational and Regression Analysis

Using listwise deletion, data of 238 participants could be entered in the regression model. Correlational analysis showed significant correlations between the General-HL index and sex and between the General-HL index and all personality traits (Table 3). The highest correlation with the General-HL index was found with Conscientiousness. Some predictor variables were significantly intercorrelated: Sex was correlated with Neuroticism, Openness to experience, and Agreeableness, while age was correlated with education, Openness to experience, and Conscientiousness (see Table 3). Education was significantly correlated with Openness to experience (Table 3). Some of the Big Five personality traits were significantly intercorrelated too: The highest correlation was found between Neuroticism and Extraversion, followed by Neuroticism and Conscientiousness and by Extraversion and Conscientiousness.

In the regression model, which was used to test our hypotheses, Neuroticism, Openness to experience, Conscientiousness, and sex were significant predictors of HL (see Table 4). The first model (sex, age, and education) was not significant, with a very small adjusted *R*^2^ of 1.7%. The second model included the Big Five personality traits and accounted for 32.2% of variance in the General-HL index, which is considered a large effect. Neuroticism (β = −0.21, *t*(229) = −2.98, *p* = 0.003), Openness to experience (β = 0.15, *t*(229) = 2.35, *p* = 0.019), Conscientiousness (β = 0.31, *t*(229) = 4.84, *p* < 0.001), and sex (β = 0.14, *t*(229) = 2.34, *p* = 0.020) were significant predictors of subjective HL (Table 4). Conscientiousness was the strongest predictor, followed by Openness to experience, Neuroticism, and finally sex (see β in Table 4). All predictors were positively associated with subjective HL, except Neuroticism (β = −0.21). Therefore, those belonging to the female sex and those with low Neuroticism, high Openness to experience, and high Conscientiousness were more likely to have better subjective HL. Extraversion, Agreeableness, age, and education were not predictive of subjective HL in the regression model (Table 4).

## 4. Discussion

Our main findings are that (i) higher Conscientiousness (β = 0.31, medium effect), lower Neuroticism (β = −0.21, small effect), and higher Openness to experience (β = 0.15, small effect) were all significantly associated with higher levels of subjective HL. In contrast, Agreeableness and Extraversion did not significantly predict subjective HL (Table 4, Model 2). (ii) Concerning sociodemographic variables, the female sex was a significant positive predictor, while age and education did not significantly predict subjective HL. (iii) Furthermore, we showed that personality dimensions contributed significantly over and above sociodemographic variables in predicting subjective HL: The first model (including only sex, age, and education) was not significant, with a very small adjusted R^2^ of 0.017, whereas the second model (also including personality dimensions) was significant, with an adjusted R^2^ of 0.322.

Based on the evidence from the existing literature, we expected Conscientiousness to have the strongest positive association with subjective HL, Neuroticism to be negatively associated with subjective HL, and Agreeableness, Extraversion, and Openness to experience to show at most positive associations of small effect sizes with subjective HL. Thus, our results on Conscientiousness and Neuroticism are very much in line with our hypotheses. Additionally, Openness to experience turned out to be significantly associated with subjective HL as well. Our results are in line with results from a recent previous study from Switzerland that found that more open individuals had higher health literacy competencies, whereas individuals with higher levels of Neuroticism had more difficulties with specific health-related tasks or situations [29]. In our study, however, Conscientiousness was the strongest predictor. This was not found in the Swiss study [29], but it aligns with results from another study on older adults from Japan that found Openness, Neuroticism, Conscientiousness, and Extraversion to be associated with health literacy [27].

Gerich and Moosbrugger (2018) stated two dimensions or factors as possible pathways to perceiving one’s own manageability of health-related tasks to be high: (a) high personal resources (e.g., self-efficacy and active coping) or (b) high trust and adherence to instructions given by medical professionals [3]. Conscientiousness has previously been positively and consistently associated with both personal resources [17,18,19] and trust and adherence [20,21,22,23]. Thus, both pathways might have acted together and might help to explain why Conscientiousness had the largest effect size of all significant associations with subjective HL. Similar explanations apply for Neuroticism (but for the reverse effect): Neuroticism has previously been (mostly consistently) negatively associated with both personal resources [17,18,19] and trust and adherence [21] and was also found to have a negative association with subjective HL in our study. Openness, as a third significant predictor, has been positively associated with both personal factors [19] and trust and adherence [20]. For Openness, however, previous results were less consistent as some studies found significant correlations and others did not, which might contribute to explaining the small effect size for Openness to experience in our study.

Apart from their association to personal resources and trust and adherence, Conscientiousness, Neuroticism, and Openness to experience have been found to predict several health outcomes to different degrees in previous research. Conscientiousness has been positively associated with both subjective assessments of one’s own health [38] (Lodi-Smith et al., 2010) and assessments by physicians and experts [39], as well as with a variety of objective health outcomes (e.g., positive influence on the self-assessed health status, blood pressure, and the number of working days (fewer days absent)) [40]. It has been shown that the likelihood of suffering a stroke or heart attack is reduced by high Conscientiousness [41], as is the likelihood of suffering from Alzheimer’s disease [42]. In addition, several authors emphasized the positive effect of Conscientiousness on longevity and lower mortality, respectively [43,44,45]. Conscientiousness has even been considered to be one of the strongest predictors of physical health in general [44,46]. Also with regard to Neuroticism, strong associations with health outcomes have been found: Neuroticism was found to have a negative influence on the self-assessed health status, blood pressure, and number of working days (more days absent) [40]. In a large study (N > 3000), Neuroticism—in addition to Conscientiousness—was identified as the personality trait with the strongest correlations to physical diseases: Skin problems, sciatica or urinary tract problems, asthma, tumors, and lung problems have been associated with higher levels of Neuroticism [46]. Furthermore, associations have been found to hypertension [47], development of obesity, metabolic syndrome [48], cardiovascular diseases [49], and mortality [50]. Concerning Openness to experience, previous research has linked high Openness to beneficial health behaviors (but there was a lower association with objective health outcomes): High levels of Openness were associated with health-related internet searches [51], with more gathering of information and less avoidance concerning future care [52]. People with high Openness also tend to have the most active healthcare decision-making style: They prefer to discuss treatment choices and to have personal control over important decisions [50]. Nevertheless, Openness to experience has not been linked to objective health outcomes to the same extent as Conscientiousness and Neuroticism.

Our findings showed no significant associations between Extraversion, Agreeableness, and subjective HL, despite their theoretical links to social support and trust in healthcare [20,21]. One possible explanation is that subjective HL relies more on personal resources like self-efficacy and active coping [19], which are more strongly associated with Conscientiousness and Neuroticism—both of which showed significant effects. Regarding the two pathways suggested by Gerich and Moosbrugger (2018) [3], our results indicate that personal resource-based manageability may be more decisive for subjective HL than trust-based manageability. However, given prior findings on the relevance of social traits, future research should examine whether specific facets of Extraversion (e.g., assertiveness) and Agreeableness (e.g., trust) contribute to subjective HL in more nuanced ways.

As mentioned before, the HLS-EU Scale has been criticized for assessing HL only subjectively [5]. Nevertheless, subjective HL has been associated with health-related outcomes [7] and was proposed to contribute to shaping health status in the sense of a self-fulfilling prophecy [3]. Thus, our results, in combination with prior research, suggest that subjective HL is a possible mediator for the numerous associations found for personality dimensions and health outcomes in previous research. This might most strongly be the case for Conscientiousness (and Neuroticism) and less for Openness to experience; this should be investigated in future studies.

Concerning sociodemographic variables, a significant association was found for the female sex and higher levels of subjective HL. This result is in line with previous findings on sex and subjective HL [4] and also matches results from another study that showed that women tend to visit their primary care provider more often than men [53]. However, it should be noted that sex only had a minor effect compared to the personality dimensions, and it is noticeable that sex was not significantly associated in the first regression model but only in the second model when personality traits were included (Table 4). This might be due to correlations between sex and personality traits: As stated in Table 3, there are significant correlations between sex and Neuroticism, Openness, and Agreeableness. Interaction effects between sex and personality could thus be of interest in future studies. In contrast to previous studies that reported an association between subjective HL and lower age and higher education [4], we did not find associations for either age or education in our study. However, it should be considered that the level of education of our sample was above the national average: In Germany, about 30% of people aged 50–54 and 16.6% of people aged 65 or older have achieved the highest school degree (advanced technical college entrance qualification/university entrance qualification) [54], whereas in our sample, 62.9% hold this qualification. Additionally, the variance in years of schooling was low. In addition to an above-average level of education, the participants of our sample also scored above the national average in terms of subjective HL, even though participants of the representative norm sample were younger (M = 46 years of age, SD = 18) than participants in our sample (M = 61.93 years of age, SD = 10.30) [4]. Nevertheless, our results match the results of a previous study that also found above-average levels of subjective HL (via the short version of HLS-EU-Q16) in older adults from East Germany [55]. Here, the authors argue that older adults may have greater exposure to health issues due to the rising prevalence of diseases in later life. Nevertheless, many of these elderly experience high levels of well-being and maintain good functional abilities despite having one or more diseases [56]. Feeling healthy, capable, and resilient despite being older (or suffering from disease) might thus even increase the ‘perception of manageability’ of one’s own health, as proposed by Gerich and Moosbrugger (2018) [3].

The aim of this study was to extend the approach of Gerich and Moosbrugger (2018) [3] in terms of how less alterable personal resources (personality dimensions) contribute to establishing an individual’s perception of his or her manageability of health-related tasks over and above sociodemographic variables (sex, age, and education). In that respect, we contributed to ruling out the discussed explanation of reverse causality (individuals may have little experience with health-related problems, which might affect both their personal health-related resources and their level of trust and adherence, thus affecting their subjective HL). Since the Big Five are usually regarded as persistent and stable concepts [57], they are less likely altered by experiences with health-related problems than personal resources (e.g., self-efficacy and coping style) or trust and adherence in medical professionals.

### 4.1. Strengths and Limitations

Some limitations should be considered: First, as indicated, our sample was not representative of older adults in Germany regarding the high level of education. This limited variance in years of schooling, which may have suppressed potential correlations between education and health knowledge. Additionally, older cohorts with the same “years of education” might differ qualitatively in health knowledge, which was not accounted for in this study. A second limitation is the cross-sectional design of this study: Even though the Big Five are usually regarded as persistent and stable concepts [57] that predict a variety of life events and outcomes, the possibility of change in personality remains [58]. Thus, only longitudinal studies may differentiate between correlational and causal relations. Furthermore, our study is limited to individuals living in Germany, which may have an impact on the generalizability of the results. However, it should be emphasized that the Big Five dimensions are considered to be universal across countries and cultures [59]; so, in terms of personality, it is likely for our findings to be applicable to other countries as well. However, future studies should investigate subjective HL in developing countries as the associations between personality and subjective HL might change when access to healthcare is more difficult.

A major strength of this study is that we extended the work by Gerich and Moosbrugger (2018) [3] to personal resources that are less prone to change and thus contribute to understanding the rationale behind subjective HL.

### 4.2. Implications

We would like to emphasize that we do not suggest that subjective HL should be considered exclusively from a singular individual level or even from a deterministic perspective: Personality dimensions being associated with subjective HL do not absolve societies from their obligation to ensure appropriate education and training in health-related issues. On the contrary, we would like to promote the idea of understanding subjective HL as a reciprocal interplay of individual and systemic factors: The emergence of high subjective HL should be considered as a comprehensive inferential process. From an organizational perspective and in terms of empowerment, it should be of interest to what extent healthcare systems enable people to manage health issues appropriately. The design and delivery of quality care must be tailored to the individual’s needs and characteristics as precisely as possible [60]. Personality does not determine subjective HL or health outcomes solely but does contribute to shaping the way people perceive the manageability of health-related tasks: Within the Health Belief Model, personality as part of the modifying variables is likewise understood to affect health-related behaviors indirectly by affecting perceived seriousness, susceptibility, benefits, and barriers [61]. Thus, when subjective HL is considered, future research should stretch the mutual interaction of personal factors (micro level) and organizational and societal factors (macro level), with personality dimensions being one of the personal factors of interest. This applies to both theoretical and practical approaches, such as tailoring well-adjusted programs or interventions to improve subjective HL. Further research can exploit the results of this study by investigating how personality traits influence health literacy across different contexts. Longitudinal studies would help clarify the causal relationship between the Big Five and health literacy over time. Additionally, research in diverse cultural and socioeconomic settings could provide insights into how these associations might change, particularly in countries with limited access to healthcare. This could enhance our understanding of how personality shapes health literacy and inform the development of more personalized health interventions.

Our results suggest that personality traits, particularly Conscientiousness, are linked to higher health literacy, indicating that health literacy interventions should be personalized. Health policies could develop tailored educational programs for different personality types, with a focus on stress reduction and self-efficacy for individuals with high Neuroticism. Healthcare providers could also implement more individualized communication strategies, such as clearer action plans for less conscientious patients and personalized digital health tools. Additionally, the findings highlight the importance of patient-centered communication. Healthcare professionals should be trained in personalized approaches, offering detailed materials for conscientious individuals and emotional support for neurotic ones, thereby enhancing the effectiveness of health interventions—something that could be increasingly implemented in the future as personalized and digital care models evolve.

## 5. Conclusions

Our study reveals that low Neuroticism, high Openness to experience, and high Conscientiousness are associated with better subjective health literacy (HL) in adults aged 50 and older. These findings highlight the importance of personality traits in shaping subjective HL. Additionally, we found that the female sex is linked to better subjective HL, emphasizing the need for further gender-specific research and more personalized interventions. We stress the importance of understanding personality dimensions as a key factor in the interplay between individual and systemic influences on subjective HL.

## Figures and Tables

**Table 1:** Participant characteristics (*n* = 238).

	Male Participants		Female Participants			Total	
Variable	*n*	%/*M* (*SD*)	*n*	%/*M* (*SD*)	*p*	*n*	%/*M* (*SD*)
**Sex ^a^**	113	47.5%	125	52.5%		238	100%
**Age**	113	62.44 (9.68)	125	61.47 (10.85)	0.469	238	61.93 (10.30)
**Education ^b^**	113	11.74 (1.95)	125	11.66 (1.87)	0.749	238	11.70 (1.91)
**Highest school degree**					0.213		
No school certificate	0	/	2	1.6%		2	0.8%
Secondary general school certificate ^c^	18	15.9%	13	10.5%		31	13.1%
Intermediate school certificate ^d^	22	19.5%	33	26.6%		55	23.2%
Advanced technical college entrance qualification/ University entrance qualification ^e^	73	64.6%	76	61.3%		149	62.9%
**Cognitive status**							
MMSE	113	29.29 (0.92)	125	29.29 (1.01)	0.974	238	29.29 (0.97)
**Depression**							
GDS	112	1.46 (1.90)	125	1.26 (1.53)	0.357	237	1.35 (1.71)
**Personality ^f^**							
Neuroticism	113	13.64 (7.11)	125	17.35 (7.13)	<0.001	238	15.59 (7.34)
Extraversion	113	28.79 (6.13)	125	29.56 (5.38)	0.301	238	29.20 (5.75)
Openness	113	28.78 (6.86)	125	31.38 (5.62)	0.001	238	30.15 (6.36)
Agreeableness	113	32.60 (5.97)	125	35.96 (4.85)	<0.001	238	34.37 (5.66)
Conscientiousness	113	36.51 (6.09)	125	36.16 (5.64)	0.647	238	36.32 (5.85)

Neuroticism, Extraversion, Openness to experience (=Openness), Agreeableness, and Conscientiousness were measured using the NEO-FFI. Significant values are printed in boldface. *M* = mean; *SD* = standard deviation; MMSE = Mini-Mental Status Examination; and GDS = Geriatric Depression Scale. ^a^ Sex: male = 0 and female = 1; ^b^ Education: Years of school; ^c^ Secondary general school certificate: “Hauptschulabschluss” in Germany, ^d^ Intermediate school certificate: “Realschulabschluss” in Germany, ^e^ Advanced technical college entrance qualification/University entrance qualification: “Fachhochschulreife/ Allgemeine Hochschulreife” in Germany, including vocational diploma and University entrance qualification in the second-chance education; and ^f^ personality: Sex differences calculated with a Bonferroni-adjusted α-level of *p* < 0.01 (0.05/5).

**Table 2:** Distribution of health literacy (HLS-EU-Q47) in the sample (*n* = 238).

	Male Participants		Female Participants				Total	
Variable	*n*	*M* (*SD*)	*n*	*M* (*SD*)	*p*	*d*	*n*	*M* (*SD*)
**General-HL index**	113	36.16 (8.11)	125	38.18 (7.78)	0.052	−0.254	238	37.22 (7.98)
**HC-HL index**	111	36.21 (8.34)	121	37.91 (7.78)	0.109	−0.211	232	37.09 (8.08)
**DP-HL index**	112	36.68 (8.77)	122	38.78 (8.42)	0.063	−0.245	234	37.77 (8.64)
**HP-HL index**	109	35.20 (8.81)	121	38.16 (8.66)	**0.011**	−0.338	230	36.76 (8.84)

HL = Health literacy, HC-HL index = Healthcare literacy index, DP-HL index = Disease prevention literacy index, HP-HL index = Health promotion literacy index. All HL indices were measured using the HLS-EU-Q47. Sex differences calculated with a Bonferroni-adjusted α-level of *p* < 0.01 (0.05/4) and significant values are printed in boldface. Sex differences for categorical variables were analyzed using *t*-tests. Cohen’s d was used as the measure of effect size.

**Table 3:** Correlations of the variables for the regression models HLS-EU-Q47 and NEO-FFI (*n* = 238).

Variable	1	2	3	4	5	6	7	8	9
**1. HLS**	-								
**2. Sex ^a^**	0.13 *	-							
**3. Age**	−0.09	−0.05	-						
**4. Education ^b^**	0.10	−0.02	−0.30 ***	-					
**5. N**	−0.39 ***	0.25 ***	0.02	−0.10	-				
**6. E**	0.43 ***	0.07	−0.11	0.09	−0.51 ***	-			
**7. O**	0.32 ***	0.21 **	−0.15 **	0.32 ***	−0.13 *	0.43 ***	-		
**8. A**	0.19 **	0.30 ***	−0.03	0.10	−0.12 *	0.28 ***	0.32 ***	-	
**9. C**	0.47 ***	−0.03	−0.17 **	0.04	−0.46 ***	0.44 ***	0.18 **	0.02	-

HLS = General health literacy index of the HLS-EU-Q47. Neuroticism (=N), Extraversion (=E), Openness to experience (=O), Agreeableness (=A), and Conscientiousness (=C) were measured using the NEO Five-Factor Inventory. ^a^ Sex: male = 0 and female = 1. ^b^ Education: Years of school. * *p* < 0.05, ** *p* < 0.01, and *** *p* < 0.001.

**Table 4:** Multiple linear regression analysis: NEO-FFI and HLS-EU-Q47 (*n* = 238).

	*B* [95% CI]	*SE (B)*	β	*p*	Correlation	
Variable					Bivariate	Partial
**Model 1**						
**Constant**	34.23 [23.64, 44.82]	5.38		**<0.001**		
**Sex ^a^**	2.00 [−0.03, 4.03]	1.03	0.13	0.053	0.13	0.13
**Age**	−0.04 [−0.14, 0.06]	0.05	−0.05	0.441	−0.09	−0.05
**Education ^b^**	0.38 [−0.18, 0.94]	0.28	0.09	0.181	0.10	0.09
***R* ^2^**	0.030 (*p* = 0.070), not significant					
**Adjusted *R*^2^**	0.017					
**Model 2**						
**Constant**	11.15 [−2.77, 25.06]	7.06		0.116		
**Sex ^a^**	2.25 [0.36, 4.15]	0.96	**0.14**	**0.020**	0.13	0.15
**Age**	0.01 [−0.07, 0.10]	0.04	0.02	0.767	−0.09	0.02
**Education ^b^**	0.07 [−0.43, 0.56]	0.25	0.02	0.790	0.10	0.02
**Neuroticism**	−0.23 [−0.38, −0.08]	0.08	**−0.21**	**0.003**	−0.39	−0.19
**Extraversion**	0.14 [−0.06, 0.33]	0.10	0.10	0.183	0.43	0.09
**Openness to experience**	0.19 [0.03, 0.35]	0.08	**0.15**	**0.019**	0.32	0.15
**Agreeableness**	0.05 [−0.12, 0.22]	0.09	0.04	0.557	0.19	0.04
**Conscientiousness**	0.42 [0.25, 0.60]	0.09	**0.31**	**<0.001**	0.47	0.31
**Δ*R*^2^**	0.315 **(*p* < 0.001)**					
**Total *R*^2^**	0.345 **(*p* < 0.001)**					
**Adjusted Total *R*^2^**	**0.322**					

Note. CI = confidence interval, SE = standard error. ^a^ Sex: male = 0 and female = 1. ^b^ Education: Years of school. Neuroticism, Extraversion, Openness to experience, Agreeableness, and Conscientiousness were measured using the NEO Five-Factor Inventory. Significant values are printed in boldface.

## Data Availability

No new data were created or analyzed in this study. Data sharing is not applicable to this article.

## Supplementary Materials

The following supporting information can be downloaded at: https://www.mdpi.com/article/10.3390/ijerph22030392/s1, Figure S1: Distribution of health literacy levels (HLS-EU Q47) in the total sample (*N* = 255) and by gender (female *n* = 133 and male *n* = 122); Table S1: Participant characteristics (total sample: *N* = 278); Table S2: Distribution of health literacy levels (HLS-EU-Q47) in the sample (total sample: *N* = 278).
