# Supplementary material for: Subjective Health Literacy and Personality in Older Adults: Conscientiousness, Neuroticism, and Openness as Key Predictors—A Cross-Sectional Study
Source: Int J Environ Res Public Health. 2025 Mar 7;22(3):392. doi: 10.3390/ijerph22030392 (PMC11942053; doi:10.3390/ijerph22030392)
Supplement: Supplementary file 1 [file ijerph-22-00392-s001.zip › Figure S1.pdf]

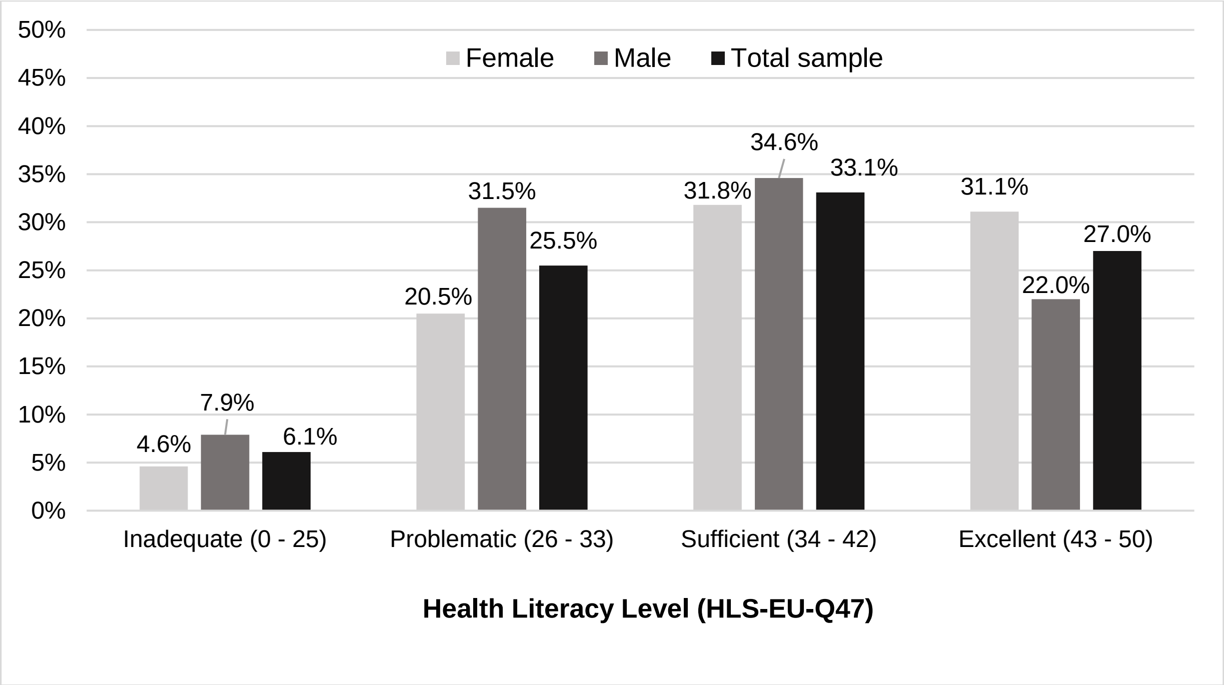

Figure S1. Distribution of Health Literacy Levels (HLS-EU Q47) in the total sample (*N* = 255) and by gender (female *n* = 133, male *n* = 122).

The classification into the levels was based on the General-HL index (score range 0-50). Missing General-HL index values in 23 of 278 participants (= 255 participants with a General-HL index).
