# Supplementary material for: Subjective Health Literacy and Personality in Older Adults: Conscientiousness, Neuroticism, and Openness as Key Predictors—A Cross-Sectional Study
Source: Int J Environ Res Public Health. 2025 Mar 7;22(3):392. doi: 10.3390/ijerph22030392 (PMC11942053; doi:10.3390/ijerph22030392)
Supplement: Supplementary file 1 [file ijerph-22-00392-s001.zip › Table S1.pdf]

Table S1. Participant characteristics (Total sample: *N* = 278).

	Male		Female			Total	
**Variable**	*n*	% / *M* (*SD*)	n	% / *M* (*SD*)	*p*	*N*	% / *M* (*SD*)
**Sex**[a]	127	45.7%	151	54.3%		278	100%
**Age**	127	62.39 (9.63)	151	61.70 (10.57)	0.572	278	62.02 (10.14)
**Education**[b]	117	11.79 (1.93)	141	11.67 (1.85)	0.634	258	11.73 (1.89)
**Highest school degree**					0.524		
No school certificate	0	/	2	1.3%		2	0.7%
Secondary general school certificate	19	15.0%	16	10.6%		35	12.6%
Intermediate school certificate[d]	25	19.7%	39	25.8%		64	23.0%
Fachhochschule/ University entrance qualification	83	65.3%	93	61.7%		176	63.3%
**Cognitive status**							
MMSE	127	29.28 (0.93)	151	29.32 (0.98)	0.721	278	29.31 (0.95)
**Depression**							
GDS	126	1.48 (1.86)	151	1.27 (1.57)	0.321	277	1.36 (1.71)
**Personality**[f]							
Neuroticism	127	13.83 (7.04)	151	17.31 (7.33)	**<.0001**	278	15.72 (7.40)
Extraversion	127	28.48 (6.04)	151	29.76 (5.72)	0.071	278	29.18 (5.90)
Openness	127	28.37 (6.67)	151	30.74 (5.95)	**0.002**	278	29.67 (6.39)
Agreeableness	127	32.40 (5.87)	151	35.91 (4.77)	**<0.001**	278	34.31 (5.57)
Conscientiousness	127	36.53 (5.89)	151	36.17 (5.73)	0.608	278	36.33 (5.80)

Neuroticism, extraversion, openness to experience (= openness), agreeableness and conscientiousness were measured using the NEO-FFI.

Significant values are printed in boldface.

M = mean; *SD* = standard deviation; MMSE = Mini-Mental-Status Examination; GDS = Geriatric Depression Scale.

[a]Sex: male = 0, female = 1; [b]Education: Years of school; [c]Secondary general school certificate: "Hauptschulabschluss" in Germany, [d]Intermediate school certificate: "Realschulabschluss" in Germany, [e]Fachhochschule/ University entrance qualification: "Abitur" in Germany; including vocational diploma and "Abitur" on the second-chance education; [f]personality: Sex differences calculated with Bonferroni adjusted α-level with $p < 0.01$ (0.05 / 5).
