# Supplementary material for: Subjective Health Literacy and Personality in Older Adults: Conscientiousness, Neuroticism, and Openness as Key Predictors—A Cross-Sectional Study
Source: Int J Environ Res Public Health. 2025 Mar 7;22(3):392. doi: 10.3390/ijerph22030392 (PMC11942053; doi:10.3390/ijerph22030392)
Supplement: Supplementary file 1 [file ijerph-22-00392-s001.zip › Table S2.pdf]

Table S2. Distribution of health literacy (HLS-EU-Q47) in the sample (Total sample: *N* = 278).

	Male		Female			Total	
**Variable**	*n*	*M* (*SD*)	*n*	*M* (*SD*)	*p*	*N*	*M* (*SD*)
**General-HL index**	122	36.02 (8.05)	133	38.07 (7.90)	0.041	255	37.09 (8.02)
**HC-HL index**	121	36.03 (8.34)	138	37.24 (8.20)	0.239	259	36.67 (8.27)
**DP-HL index**	121	36.51 (8.74)	130	38.67 (8.50)	0.048	251	37.63 (8.67)
**HP-HL index**	117	35.11 (8.70)	132	37.83 (8.87)	0.016	249	36.55 (8.88)

HL = Health literacy, HC-HL index = Healthcare Literacy Index, DP HL index = Disease Prevention Literacy Index, HP-HL index = Health Promotion Literacy index.

All HL indices were measured using the HLS-EU-Q47.

Sex differences calculated with Bonferroni adjusted α-level with $p < 0.01$ (0.05 / 4) and significant values are printed in boldface.
